# Biocompatibility evaluation of bioprinted decellularized collagen sheet implanted in vivo cornea using swept‐source optical coherence tomography

**DOI:** 10.1002/jbio.201900098

**Published:** 2019-07-23

**Authors:** Jaeseok Park, Kyoung‐Pil Lee, Hyeonji Kim, Sungjo Park, Ruchire E. Wijesinghe, Jaeyul Lee, Sangyeob Han, Sangbong Lee, Pilun Kim, Dong‐Woo Cho, Jinah Jang, Hong K. Kim, Mansik Jeon, Jeehyun Kim

**Affiliations:** ^1^ School of Electronic Engineering, College of IT Engineering Kyungpook National University Daegu South Korea; ^2^ Department of Ophthalmology, School of Medicine Kyungpook National University Daegu South Korea; ^3^ Bio‐Medical Institute Kyungpook National University Hospital Daegu South Korea; ^4^ Department of Mechanical Engineering Pohang University of Science and Technology Pohang South Korea; ^5^ Laser Application Center, Institute of Advanced Convergence Technology Kyungpook National University Daegu South Korea; ^6^ Department of Biomedical Engineering, College of Engineering Kyungil University Gyeongsan South Korea; ^7^ Institute of Biomedical Engineering, School of Medicine Kyungpook National University Daegu South Korea; ^8^ Department of Creative IT Engineering Pohang University of Science and Technology Pohang South Korea; ^9^ School of Interdisciplinary Bioscience and Bioengineering Pohang University of Science and Technology Pohang South Korea

**Keywords:** biocompatibility, bioprinted collagen sheet, corneal implant, noninvasive monitoring, optical coherence tomography

## Abstract

Corneal transplantation by full‐thickness penetrating keratoplasty with human donor tissue is a widely accepted treatment for damaged or diseased corneas. Although corneal transplantation has a high success rate, a shortage of high‐quality donor tissue is a considerable limitation. Therefore, bioengineered corneas could be an effective solution for this limitation, and a decellularized extracellular matrix comprises a promising scaffold for their fabrication. In this study, three‐dimensional bioprinted decellularized collagen sheets were implanted into the stromal layer of the cornea of five rabbits. We performed in vivo noninvasive monitoring of the rabbit corneas using swept‐source optical coherence tomography (OCT) after implanting the collagen sheets. Anterior segment OCT images and averaged amplitude‐scans were acquired biweekly to monitor corneal thickness after implantation for 1 month. The averaged cornea thickness in the control images was 430.3 ± 5.9 μm, while the averaged thickness after corneal implantation was 598.5 ± 11.8 μm and 564.5 ± 12.5 μm at 2 and 4 weeks, respectively. The corneal thickness reduction of 34 μm confirmed the biocompatibility through the image analysis of the depth‐intensity profile base. Moreover, hematoxylin and eosin staining supported the biocompatibility evaluation of the bioprinted decellularized collagen sheet implantation. Hence, the developed bioprinted decellularized collagen sheets could become an alternative solution to human corneal donor tissue, and the proposed image analysis procedure could be beneficial to confirm the success of the surgery.

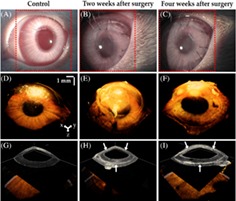

## Graphical abstract

Three‐dimensional bioprinted decellularized collagen sheets were implanted into the stromal layer of the cornea of five rabbits. After implanting the collagen sheets, the rabbit corneas were monitored biweekly in vivo with a swept‐source optical coherence tomography system to assess the biocompatibility of the bioprinted implantation for 1 month through corneal thickness measurements. The small variation in the averaged corneal thickness along with evidence from hematoxylin and eosin‐stained histology images shows the potential use of the biocompatible collagen sheet.

## 1 INTRODUCTION

The cornea is a transparent nonvascular tissue located in the outermost layer of the eye and plays a significant role in the visual function by refracting and transmitting light 1. Irreversible corneal damage can lead to vision loss and corneal transplantation is the only accepted treatment to avoid corneal blindness 2. Although corneal transplantation has been performed for over 100 years, there are some critical problems to this procedure, including graft rejection, graft failure and a lack of donors 3, 4, 5. To solve these problems, the implantation of an artificial cornea, known as a keratoprosthesis, has been introduced and is widely performed 6, 7, 8, 9, 10, 11. A keratoprosthesis is considered as an alternative treatment when corneal transplantation has failed or in cases of severe limbal epithelial deficiency with dissipated epithelial regenerative capacity after conditions such as chemical burns, Stevens‐Johnson syndrome and ocular cicatricial pemphigoid. However, it is a prosthetic device that is made of synthetic material and causes some serious complications, including persistent epithelial defects, prosthetic extrusion, retroprosthetic membrane formation and endophthalmitis. Thus, from a clinical perspective, bioengineered corneal substitutes using biocompatible materials are needed to overcome the shortcomings of corneal allografts.

Decellularized extracellular matrix (ECM) scaffolds derived from tissues have been effectively used in human clinical applications. The composition of the ECM varies according to the type of tissue as it exhibits tissue‐specific directed differentiation and the stem cells mature into a specific lineage. Therefore, the cells in the engineered tissue are more likely to mature if they are given an ECM environment that is similar to the original tissue and organ (eg, heart 12, skeletal muscle 13, dermis 14, lung 15 and liver 16). In addition, three‐dimensional (3D) bioprinting is a promising technology for building patient‐specific complex living structures in 3D via the direct deposition of biomaterials, biomolecules and cells 17. Likewise, 3D bioprinting using an ECM with decellularized corneal tissue offers a new strategy for the fabrication of bioengineered cornea and in particular, could be applied in the fabrication of patient‐specific corneas that have different curvatures or thicknesses. Thus, the implantation of patient‐specific 3D bioprinted corneas made of ECM components could replace existing treatments. Fabricated bioengineered corneal substitutes are generally evaluated using histologic examinations in experimental animal models 18. The fabricated cornea is implanted into the eye of an experimental animal for 2 or 4 weeks, and then the cornea of the animal is extracted and evaluated. However, hematoxylin and eosin (H&E) staining is not suitable for evaluating a fabricated cornea during continuous research, especially in monitoring studies, thus a noninvasive and high‐resolution system should be considered for evaluating the fabricated corneas.

Optical coherence tomography (OCT) is a real‐time noninvasive, noncontact biomedical inspection technique 19, a mainstream technology that has been widely applied in medical fields, such as ophthalmology 20, dentistry 21, dermatology 22, neurology 23, 24 and otolaryngology 25, 26. Because OCT operates on the basis of near‐infrared light, there is no risk of invasion and radiation. Moreover, the high‐resolution imaging capability has already led to applications involving corneal imaging for conditions, such as keratoconus 27, corneal opacity 28, microbial keratitis 29 and bullous keratopathy 30. Therefore, OCT can be applied for the monitoring of structural changes in the surgical region of the cornea.

In this study, we performed the noninvasive monitoring of 3D bioprinted decellularized collagen sheet (3D‐BDCS)‐implanted rabbit corneas in vivo using SS‐OCT. The main objective of the study was to confirm the biocompatibility of 3D‐BDCS by evaluating the corneal morphological change in the monitored region and by a histological analysis. 3D‐BDCS was fabricated using a 3D bioprinting technique with decellularized corneal ECM materials. The 3D‐BDCS implantation was performed on five rabbits, followed by 1 month of monitoring. As far as we are aware, this study is the first demonstration of OCT‐based in vivo monitoring of implanted 3D‐BDCS corneas.

## 2 MATERIALS AND METHODS

### 2.1 Fabrication of 3D‐BDCS

3D‐BDCSs were fabricated via a 3D cell bioprinting technique using decellularized corneal ECM containing differentiated corneal stromal cells. The corneal ECM was prepared using a decellularization process. The corneal stromal tissues were dissected from bovine eyeballs (purchased from a slaughterhouse in Gigye‐myeon, Buk‐gu, Pohang, Gyeongsangbuk‐do, South Korea) and washed with phosphate‐buffered saline (PBS) solution supplemented with penicillin (100 U mL^−1^) and streptomycin (0.1 mg mL^−1^). The stromal tissues were stirred in 0.5% Triton X‐100 (99.9% purity, Biosesang, 697 Pangyo‐ro, Bundang‐gu, Seongnam‐si, Gyeonggi‐do, South Korea) solution supplemented with 20 mM ammonium hydroxide (NH_4_OH; 4.98 N, Sigma‐Aldrich, St. Louis, Missouri). After being stirred for 4 hours, the tissues were treated with hypotonic Tris hydrochloride (Tris–HCl, pH 7.4, Biosesang) buffer solution for 24 hours, followed by treatment with 10 mM Tris–HCl containing 1% (v/v) Triton X‐100 for the next 24 hours at 37°C. Afterward, the tissues were immersed in PBS solution for 48 hours and were then sterilized with 1% peracetic acid (32 wt% in dilute acetic acid, Sigma‐Aldrich) solution in 50% ethanol for 10 hours. After the sterilization process, the samples were rinsed with PBS solution and ultrapure water. When the decellularization process was finished, the decellularized corneal ECM samples were lyophilized overnight and then crushed into a fine powder using liquid nitrogen and a milling machine. A 0.2 g quantity of decellularized corneal ECM powder was digested in 10 mL of 0.5 M acetic acid (Merck, Kenilworth, New Jersey) solution supplemented with 0.02 g pepsin (Sigma‐Aldrich) for 3 days. After complete digestion of the decellularized corneal ECM, the solution was filtered through a fine mesh with a pore size of 100 μm and neutralized to pH 7.0–7.4 with 10 M sodium hydroxide (NaOH, Sigma‐Aldrich) solution for cell culturing.

**Figure 1:**
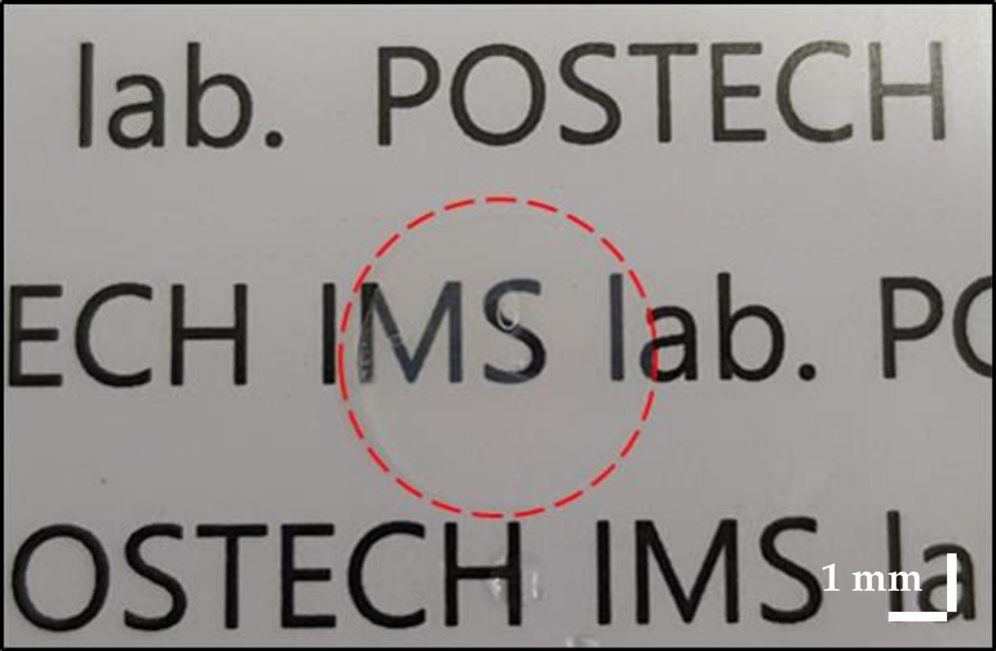
Photograph of the fabricated three‐dimensional bioprinted decellularized collagen sheet

To fabricate 3D‐BDCS, the decellularized corneal ECM hydrogel encapsulating the differentiated keratocytes 31 was printed using a microextrusion dispensing system‐equipped (Nano master SMP‐III, Musashi Engineering, Tokyo, Japan) laboratory 3D printer using the following parameters: feed rate = 130 mm min^−1^, discharge rate = 0.0024 mm s^−1^, nozzle diameter = 0.29 mm and temperature = 4°C 32. The differentiated keratocytes were taken from human turbinate‐derived mesenchymal stem cells (hTMSCs) prepared in the same manner as has previously been described in detail 28. In brief, hTMSCs were obtained from the Catholic University of Korea, St. Mary's Hospital and cultured in normal Dulbecco's modified Eagle's medium (Gibco, Gaithersburg, Maryland) containing 10% (v/v) fetal bovine serum (Gibco) and 1% (v/v) penicillin/streptomycin (Sigma‐Aldrich) at 37°C in a humidified 5% CO_2_ atmosphere. In passage 2, normal medium was replaced with differentiation medium containing 10 ng mL^−1^ keratinocyte growth factor/epidermal growth factor for 1 day to obtain the differentiated keratocytes. The printed 3D‐BDCS, which was 5 mm in diameter and 150 μm in thickness, was cross‐linked in an incubator for 30 min. Afterward, the 3D‐BDCS was cultured in the differentiation medium for 4 weeks before being implanted. The fabricated 3D‐BDCS is shown in Figure 1.

### 2.2 The in vivo preparation of the rabbits

The animal experiment was performed at Daegu‐Gyeongbuk Advanced Medical Innovation Foundation (DGMIF) (80 Cheombok‐ro, Dong‐gu, Daegu 41 061, South Korea). This study was approved by the Institutional Animal Care and Use Committee of DGMIF, was performed in accordance with protocol DGMIF‐17080801‐00 and followed the Association for Research in Vision and Ophthalmology's animal use guidelines. Five healthy New Zealand white rabbits (male, 8 weeks old, average body weight around 2 kg) were used. 3D‐BDCSs were implanted into the stromal layer of the left corneas of two rabbits (rabbits #1 and #2) and the right corneas of the remaining three rabbits (rabbits #3, #4 and #5). The surgery was performed under anesthesia using a dose of ketamine (15 mg mL^−1^) and Rompun (5 mg mL^−1^). A three‐quarter‐circle incision 6 mm in diameter was made using a crescent knife (Alcon, Fort Worth, TX). The 3D‐BDCSs were inserted into the three‐quarter‐circle incision. Three‐quarter lamellar dissections were made using a crescent knife (Alcon), then the anterior corneal flap was lifted and the 3D‐BDCS was transplanted onto the corneal stromal bed. The incised site was then covered with 3D‐BDCSs and sutured with 10‐0 Ethilon nylon. The eyes operated upon were treated with eye drops containing polymyxin B, neomycin and dexamethasone (Forus, Samil Pharmaceutical Co., Ltd., 155 Hyoryeong‐ro, Seocho‐gu, Seoul, South Korea) twice a day for 2 weeks.

To undergo the experiment, the rabbits were injected intramuscularly with a mixture of ketamine (15 mg mL^−1^) and Rompun (5 mg mL^−1^), placed carefully on the sample stage and kept for 10 minutes prior to the operation. To obtain OCT images free of microdust particles, we used physiological saline to remove foreign substances, dust particles and eyebrows. Considering the rabbits' health and the DGMIF protocol, the experiments were performed with a two‐week interval. Four weeks after surgery, the rabbits were euthanized.

### 2.3 Configuration of the swept‐source OCT system

**Figure 2:**
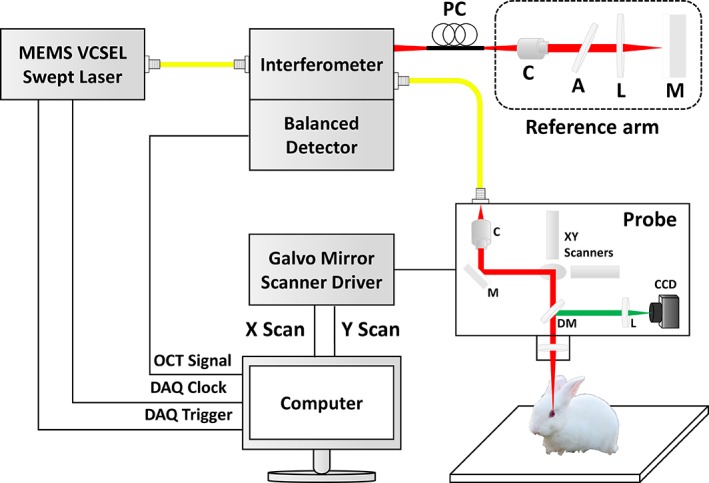
A schematic of the swept‐source optical coherence tomography (SS‐OCT) system (A, attenuator; C, collimator; CCD, charge‐coupled device; DAQ, data acquisition; DM, dichroic mirror; Galvo, galvanometer; L, lens; M, mirror; MEMS, microelectromechanical systems; OCT, optical coherence tomography; PC, polarization controller; VCSEL, vertical cavity surface emitting laser)

A schematic of the SS‐OCT system (OCS1310V1, Thorlabs, Inc., Newton, New Jersey) used in this study is shown in Figure 2. The commercial system consists of an imaging module, a swept‐source engine with a 1310 nm wavelength range and a bandwidth of >97 nm (−10 dB cutoff point) and a handheld probe. The average laser output is >20 mW; the axial scan rate and coherence length were 100 kHz and >50 nm, respectively. The experimental transverse resolution is 25 μm, and the axial resolution is <16 μm/12 μm (air/water). To obtain 2D OCT images, we set the field of view (FOV) to 10 × 4.35 mm for a total pixel count of 1000 × 435. Similarly, the volumetric images were obtained with an FOV of 10 × 10 × 4.35 mm and a pixel count of 500 × 500 × 435. The refractive index utilized was 1.38, which was the same as used in a previous report 33. Acquisition times for 2D and 3D images were measured as 0.017 and 6 seconds, respectively. Throughout the experiment, room temperature and humidity were maintained at 24 ± 1°C and 45 ± 5%, respectively.

### 2.4 Depth‐intensity profiling algorithm

**Figure 3:**
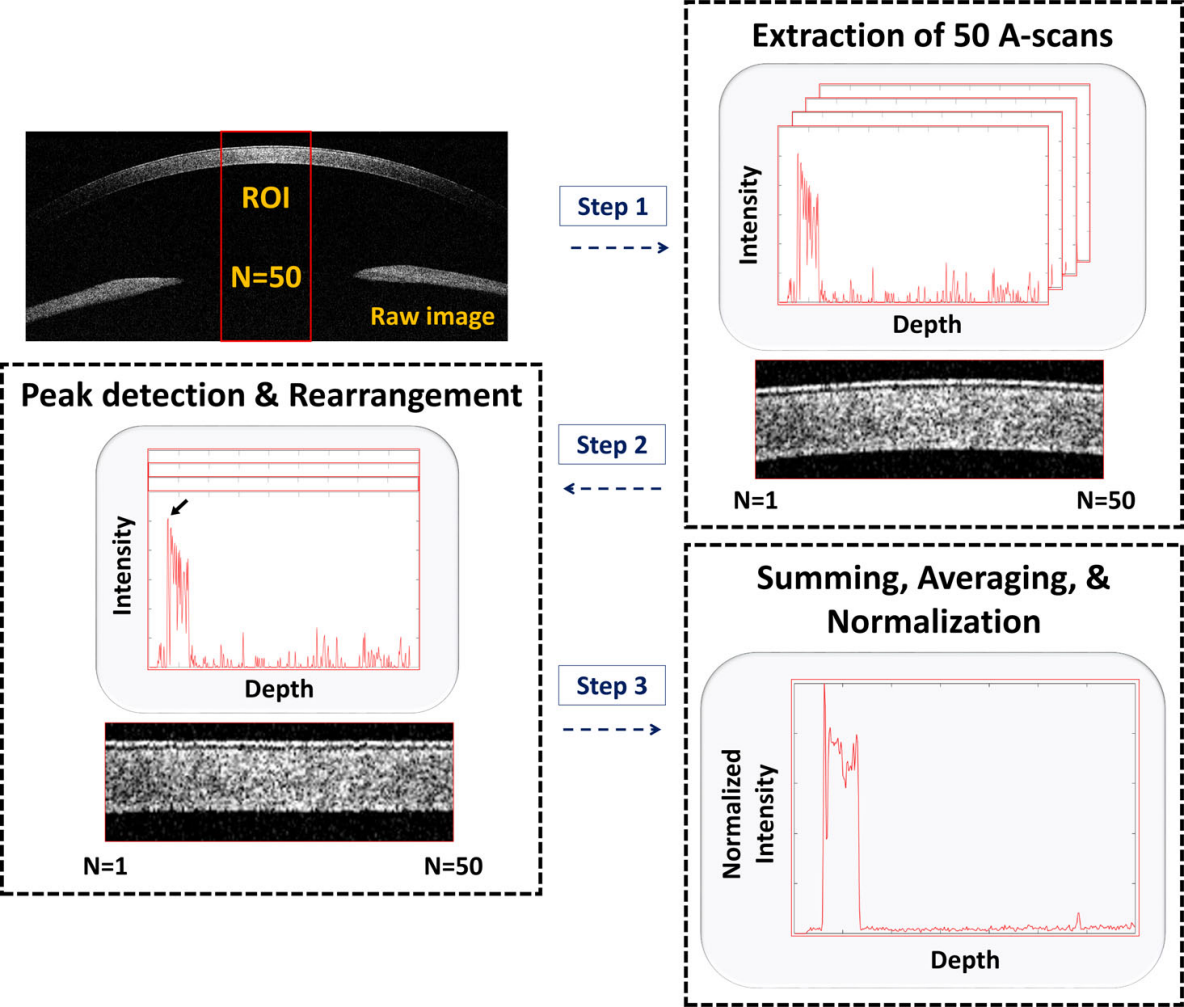
A flow chart representation of the depth‐intensity profile algorithm developed (A‐scans, amplitude scans; ROI, region of interest)

As a depth‐intensity profile analysis based on amplitude scans (A‐scans) is efficient for confirming the information on different layers along the axial direction, we developed a depth‐intensity profile algorithm using MATLAB R2018a (MathWorks, Inc., Natick, Massachusetts). However, because of the nonflat physiological nature and the anatomical structure of the cornea, a flattening function was included to rearrange each of the A‐scans. Figure 3 provides a flow chart showing the main steps of the post‐processing depth‐intensity profile algorithm developed. Once the initial cross‐sectional OCT image is loaded into the program, the region of interest (ROI) is selected to remove the speckle noise. In this study, 50 consecutive A‐scans (constant for all the acquired images) were used as the ROI. In step 2, maximum peak intensity points were detected (indicated by the black arrow) and rearranged to match the peak intensity index in all of the A‐scan profiles to flatten the image; this could be confirmed through the flat corneal image in step 2. Next, all of the A‐scan profiles of the ROI were summed and divided by the ROI range to obtain a single averaged A‐scan profile. Finally, the averaged A‐scan profile was divided by the maximum value in the averaged A‐scan profile to obtain a normalized A‐scan profile. By utilizing this method, we can confirm that unnecessary noise was removed and we could distinguish the intensity information along the axial depth.

### 2.5 The OCT image processing algorithm for the corneal thickness measurement

**Figure 4:**
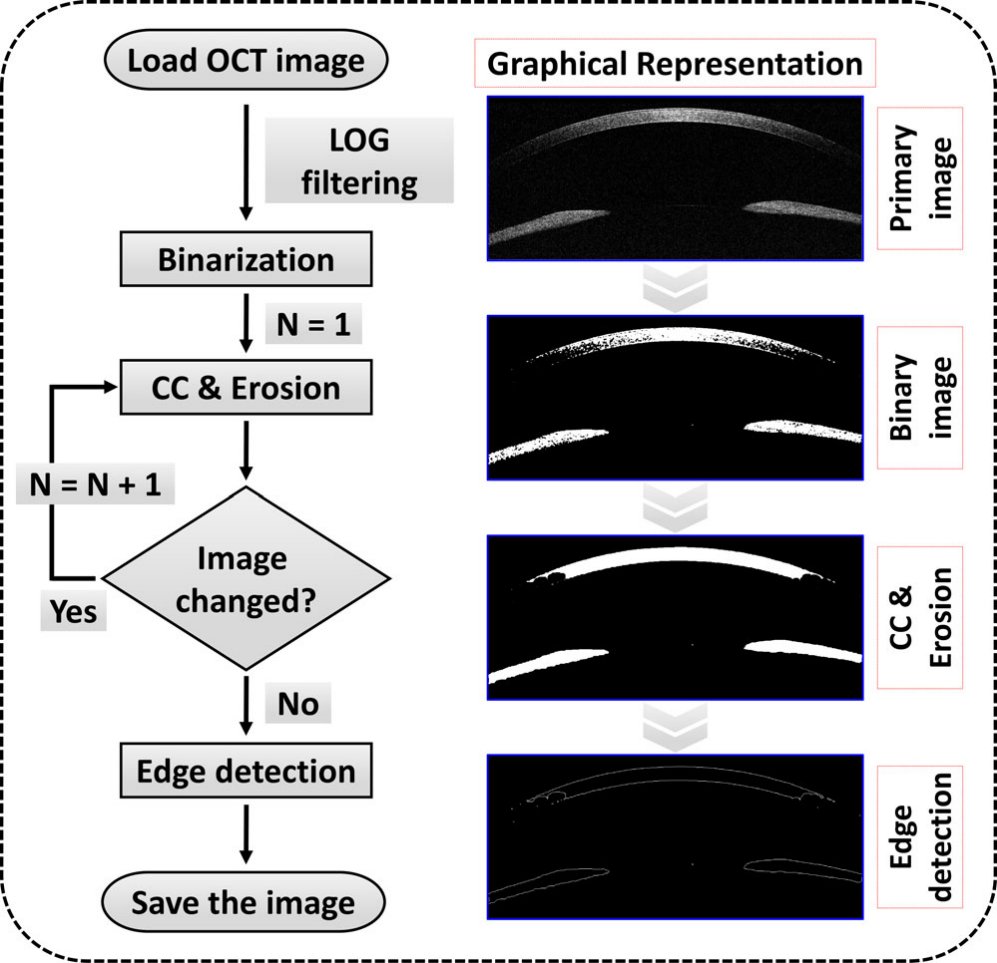
A flow chart representation of the proposed image processing technique for measuring the corneal thickness (CC, connected‐component; LOG, Laplacian of Gaussian)

Corneal thickness measurement is a major element for assessing corneal health and condition to form a diagnosis 34, 35, 36. Figure 4 shows a flow chart representation of the algorithm developed for measuring corneal thickness. Image processing was performed to obtain a clear visualization of distinguishable upper and lower corneal boundaries, as is shown in the figure. As the approximate upper and lower boundaries are difficult to distinguish, the scattering noise of the cross‐sectional OCT image was removed using the Laplacian of Gaussian (LOG) filtering technique 37. Second, the grayscale image thus obtained was converted to a binary image using Otsu's method which automatically determines the threshold in the image 38. Next, to facilitate boundary detection, connected components 39 and erosion 40 (CC & Erosion) methods were applied and repeated to fill the empty areas in the cornea until the image no longer changed. Last, we obtained clear upper and lower boundaries of the cornea using the Sobel edge detection method 41. To measure the corneal thickness in the obtained image, we found the shortest path between the upper and lower boundaries of the cornea by constructing a tangent and its perpendicular lines. We stored the pixel location at the point where the perpendicular line intersected the upper and lower boundaries. The distance between the two stored values was calculated using the distance formula between two points. The calculated value was multiplied by the pixel resolution value of 10 μm to obtain the corneal thickness.

### 2.6 Tissue preparation and histological analysis

To evaluate the corneas through histological examination, both eyes (*oculus uterque*) were extracted 4 weeks after surgery. The rabbit corneas were fixed with 4% paraformaldehyde in PBS for 24 hours. The samples were dehydrated in a series of ethanol solutions from 70% to 100% ethanol, cleared with xylene and mounted in paraffin. Slides were prepared in 6‐μm‐thick sections. Before being stained, the sections were dewaxed and rehydrated. The slides were stained with H&E. The images were obtained with an Eclipse 80i microscope (Nikon, Minato, Tokyo, Japan).

## 3 RESULTS AND DISCUSSION

### 3.1 Morphological evaluation of the rabbit eye samples prior to surgery

**Figure 5:**
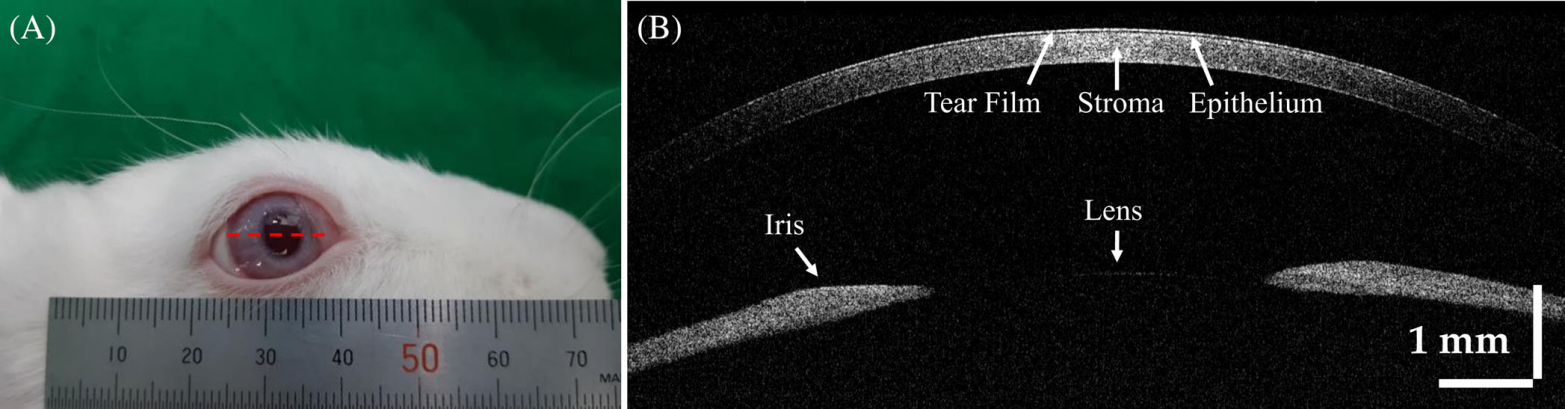
(A) Photograph of a rabbit's eye used in the experiment. (B) Cross‐sectional OCT image of (A) using the red dashed line as the scanning position

Figure 5A is a representative photograph of the rabbit eye model used in this study. The exact scanning position used in acquiring the cross‐sectional OCT images is indicated with a red dashed line. In Figure 5B, the outermost tear film is observed as a high‐intensity thin layer of the cornea. Similarly, the low‐intensity thin layer represents the epithelium. We can also confirm the stroma which occupies most of the cornea. A centered aiming beam of 660 nm and a visual camera were used to more conveniently obtain the images.

### 3.2 Comparison of the cross‐sectional OCT images with the depth‐intensity profiles

**Figure 6:**
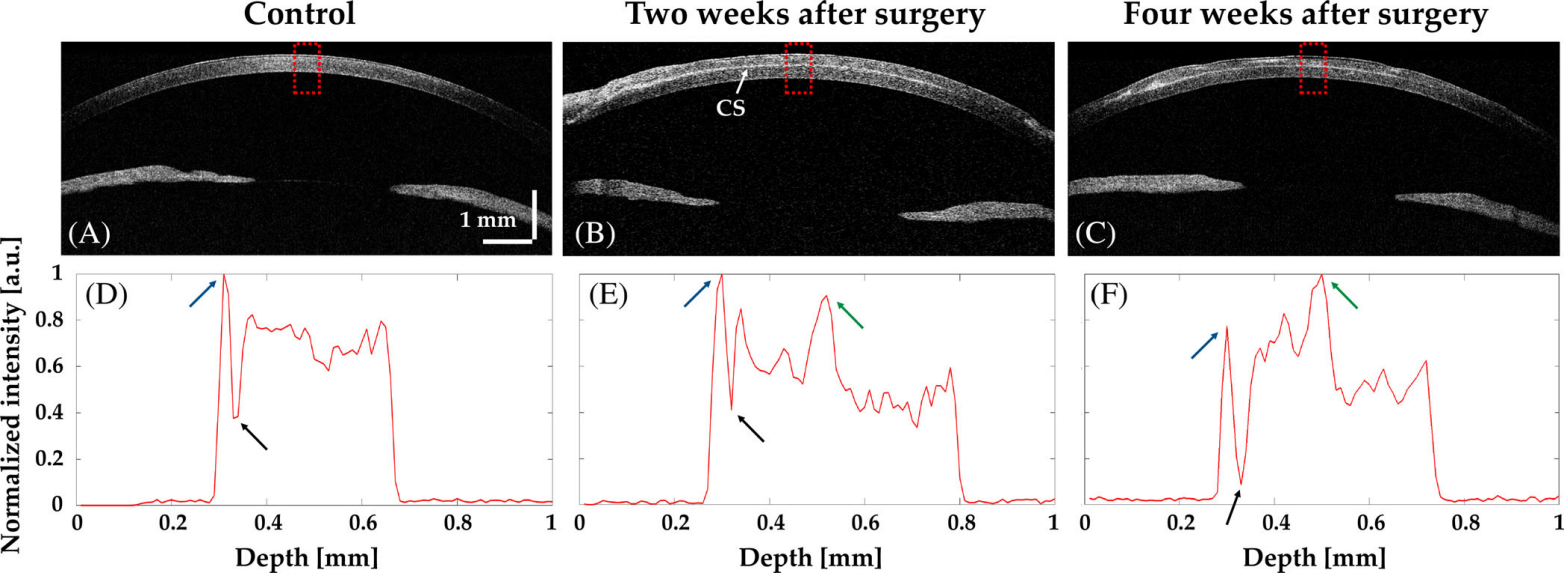
Cross‐sectional OCT images (A, B, C) and the corresponding depth‐intensity profile analyses (D, E, F) for the control (before surgery), and 2 and 4 weeks after surgery, respectively. The depth‐intensity profile analyses correspond to the regions indicated by the red dotted square in (A), (B) and (C), respectively. CS, 3D‐BDCS. The blue, black and green arrows indicate tear film, epithelium and a layer of BDCS, respectively

Figure 6 shows a comparison of cross‐sectional OCT images and depth‐intensity profile analyses for the control (before surgery), and 2 and 4 weeks after surgery. The red dotted square indicates the position analyzed in the corresponding OCT image. The intensity shown in each graph provides a relative representation of the quantitative value of the scattered signal obtained from the sample. In Figure 6D‐F, the first vertex (blue arrow) represents the tear film information. Similarly, we can see the epithelium (black arrow). The intensity peak corresponding to the 3D‐BDCS layer is clearly distinguishable in Figure 6E,F and is indicated by a green arrow. These results reveal that OCT is capable of obtaining quantitative morphological evaluations as well as performing continuous monitoring of microstructures.

### 3.3 Comparison of volumetric OCT images with orthogonal section slices

**Figure 7:**
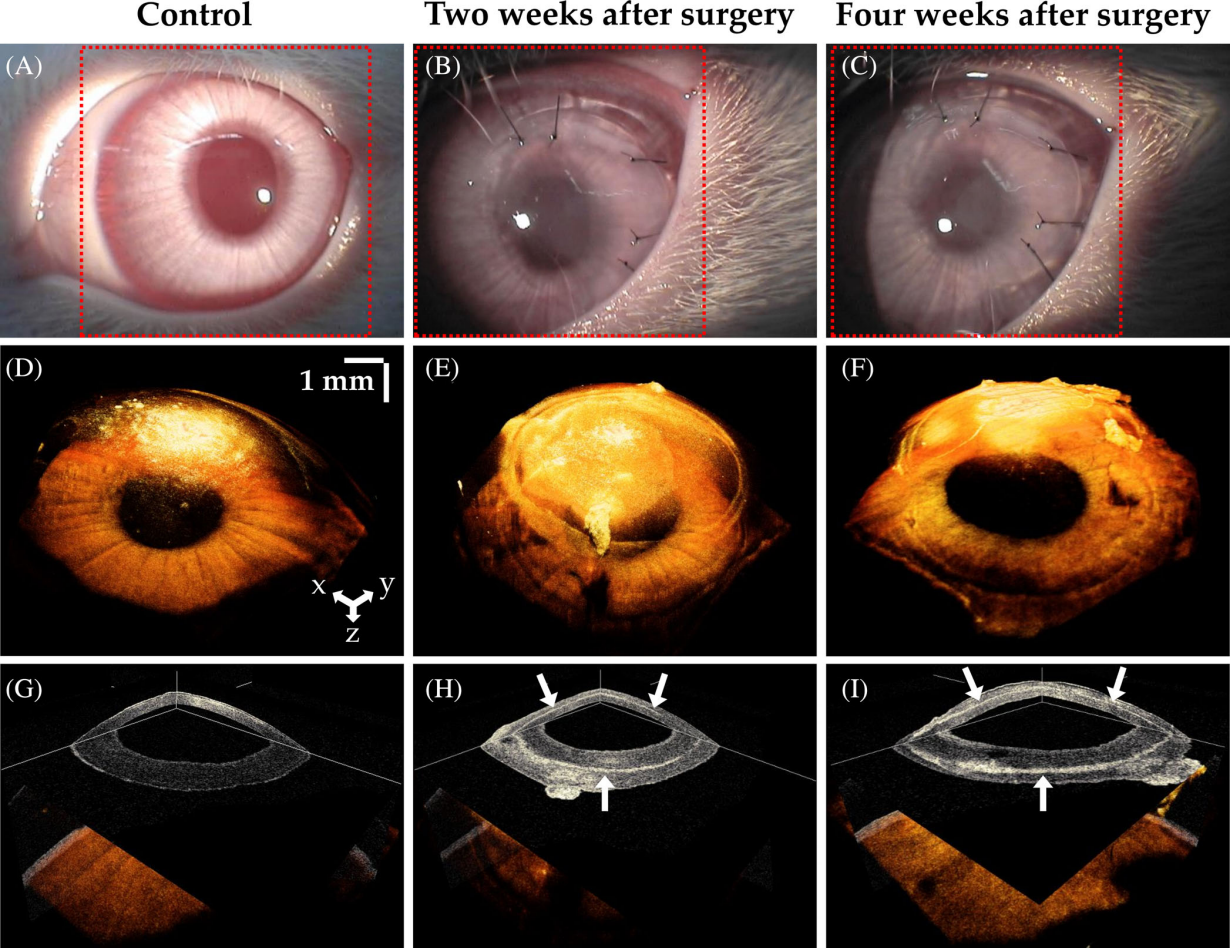
Slit‐lamp microscopic images of a rabbit's eye: (A) the control, (B) 2 weeks after surgery and (C) 4 weeks after surgery. (D), (E) and (F) are the corresponding 3D‐rendered images, and (G), (H) and (I) are the orthogonal section slice OCT images corresponding to (D), (E) and (F), respectively. Red dotted square: area of the 3D scan

The volumetric OCT images of the rabbit corneas were obtained by combining adjacent 2D OCT images using volumetric rendering software. Figure 7A‐C shows close‐up photographs of a rabbits' eyes using a slit‐lamp microscope for the control, and 2 and 4 weeks after surgery, respectively, while Figure 7D‐F presents volumetric 3D‐rendered OCT images with a red dotted square showing the area scanned by combining 500 contiguous 2D OCT images. In Figure 7B,C, we can see that the 3D‐BDCS was implanted in the eye, although the position where the 3D‐BDCS had been implanted could not be identified. Through the orthogonal section slice images shown in Figure 7G‐I in the X‐Y, X‐Z and Y‐Z directions, we can confirm that the 3D‐BDCS was correctly implanted noninvasively in the middle of the cornea (white arrow). Therefore, the OCT system could be efficiently utilized for determining the surgical success of the implantation of 3D‐BDCS in the corneas of the rabbits.

### 3.4 Evaluation of 3D‐BDCS biocompatibility with corneal thickness quantifications

**Figure 8:**
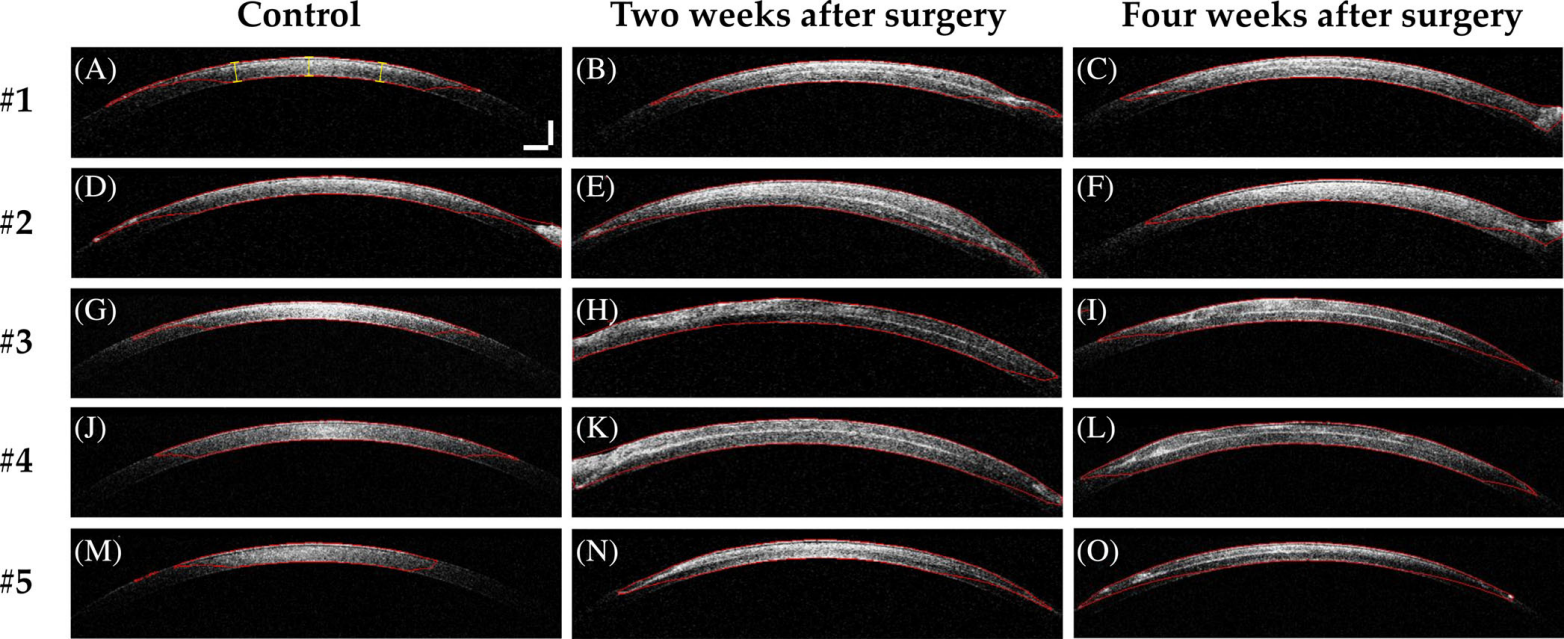
2D OCT images of samples with detected corneal boundaries (marked as a red line) for the control (A, D, G, J, M), 2 weeks after surgery (B, E, H, K, N), and 4 weeks after surgery (C, F, I, L, O) for rabbits #1‐#5, respectively. Scale bar: 500 μm

We further evaluated the morphological changes in the 3D‐BDCS‐implanted corneal region quantitatively to confirm the biocompatibility of the 3D‐BDCS with the rabbits' corneas. The algorithm we developed (described in section 2.5) was used for these quantifications. All of the corneal boundaries generated using the algorithm are highlighted in red in Figure 8. The yellow lines in Figure 8A were used as cursors for measuring the fluctuation in thickness between the two boundary lines in each cross‐section shown in Figure 8A‐O. The distance between the yellow cursors was approximately 150 μm. Thirty values for each 10 OCT image set in Figure 8A‐O were used to enhance the accuracy of the corneal thickness measurements. The measurement values are summarized in Table 1.

**Table 1:** Corneal thickness statistics for rabbits #1 to #5

	#1	#2	#3	#4	#5	Total average
Control						
Mean	426.7	434.2	427.4	424.9	438.5	430.3
SD	5.9	9.2	6.1	3.4	4.7	5.9
Min.	418.9	421.3	418.7	420.7	430.1	421.9
Max.	437.0	450.6	435.0	429.1	445.4	439.4
2 weeks						
Mean	560.1	641.4	605.2	632.6	553.4	598.5
SD	10.7	28.3	7.5	4.3	8.0	11.8
Min.	541.6	600.3	592.0	627.4	538.4	579.9
Max.	571.3	677.1	613.7	639.0	562.0	612.6
4 weeks						
Mean	524.1	580.8	597.0	592.7	527.8	564.5
SD	7.4	28.9	8.5	9.1	8.8	12.5
Min.	512.6	542.7	588.0	577.2	511.8	546.5
Max.	533.4	623.3	610.0	606.0	540.9	582.7

*Note*: Units in μm.

Abbreviations: Max., maximum value; Min., minimum value; SD, standard deviation.

Using Figure 8 and Table 1, we analyzed and confirmed the mean values of all the in vivo samples; the corneal thickness expanded (from 430.3 to 598.5 μm) owing to effects of the implantation surgery as well as the thickness of the 3D‐BDCS. Similarly, at 4 weeks, the mean value was calculated as 564.5 μm. Based on these values, we determined that the mean corneal thickness value decreased by around 5.7% from 2 to 4 weeks. Therefore, the quantitative values obtained show that the corneal thickness initially increased owing to the effects of the surgery and then gradually decreased. In other words, the corneal thickness decreased as the edema decreased. Thus, it was assumed that the 3D‐BDCS did not affect the stromal layer. Moreover, transparent corneas are a prerequisite for good vision and the endothelium has the active function of keeping the water content of the tissue regulated. As this function decreases, the extracellular water content of the tissue increases and corneal thickness is increased, resulting in corneal edema whereby light passing through the cornea is scattered and eyesight is impaired. Therefore, central corneal thickness is a sensitive indicator of corneal health. To evaluate the function of a bioengineered cornea as a corneal substitute, it is necessary to examine its thickness and morphology through in vivo experiments. Inspection methods that can simultaneously evaluate thickness along with the morphological status of the cornea have been scarce. Thus, we performed noninvasive monitoring of bioengineered cornea in rabbit corneas in vivo using swept‐source OCT (SS‐OCT), where the main objective of the study was to confirm the biocompatibility by evaluating the corneal thickness and morphological change in the monitored region.

The bioengineered corneal substitutes used in this experiment were fabricated using a bioprinting technique followed by a crosslinking procedure. Although crosslinking was used to make the constructs hard, this was not mechanically sufficient to resist the pressure inside the pocket, so we expected them to become half of their original thickness after transplantation. Nevertheless, we obtained better results than we expected and they tolerated biodegradation well.

**Figure 9:**
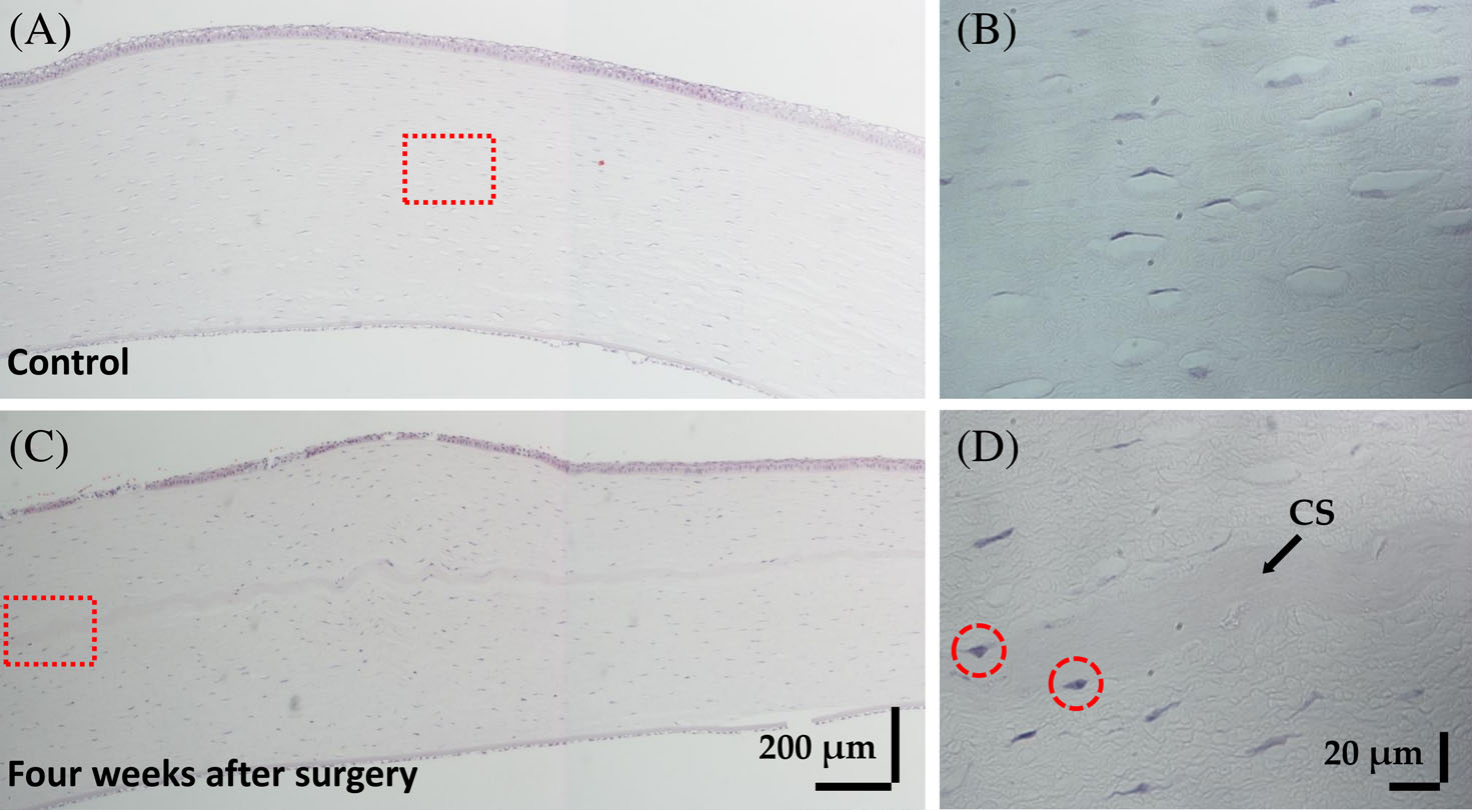
Representative images of hematoxylin and eosin (H&E) staining showing the histology of the rabbit corneas: (A) the control (the cornea without 3D‐BDCS implantation) and (C) 4 weeks after surgery. (B) and (D) are enlargements of the areas in the red dotted squares shown in (A) and (C), respectively. CS: 3D‐BDCS, Red dotted circle: inflammatory cell

We performed H&E staining to evaluate the biocompatibility of 3D‐BDCS. Figure 9 shows images of the H&E staining conducted to evaluate the immune response. Because there was no preoperative histology for the same eye, the opposite eye without the 3D‐BDCS implant was evaluated as a control. Figure 9A shows H&E staining of the opposite cornea and Figure 9C shows that for implanted one. Figure 9B,D are enlargements of the areas in the red dotted squares shown in Figure 9A,C, respectively. No inflammatory cells were found in Figure 9B. In Figure 9D, inflammatory cells (red‐dashed circles) were occasionally observed in the vicinity of the 3D‐BDCS. Nevertheless, T lymphocytes, which play a crucial role in cell‐mediated immunity 18, 42, were not observed.

After transplantation, the epithelium around the incised area was destroyed, but within a few days, the entire ocular surface had stabilized. The altered epithelium seems to have been broken during the histologic processing. In general, infiltrated inflammatory cells can be distinguished by H&E, which is also the case for the cornea 43. As shown in Figure 9, the inflammatory cells are more intensely stained with hematoxylin than the stroma, endothelial, and epithelial cells and also have a very small distribution of cytoplasm. In addition to this, the round shape of the nucleus is also indicative of inflammatory cells and makes them distinct from stromal cells. There are a number of different types of inflammatory cells (eg, granulocytes [basophils, neutrophils, and eosinophils], macrophages, monocytes and T cells). As shown, there were very few macrophages and monocytes, and T cells identified by a dark stained small dot were not observed. Hence, it is evident that some inflammatory cell types (including T cells) were not present and the remaining ones were sufficiently classified by H&E, thus we considered that an immunostaining method was unnecessary. Indeed, we have already carried out an analysis of inflammatory cells 44, 45, 46. Consequently, after considering all of the H&E stained images, we can confirm that the 3D‐BDCS associated well with the stromal layer. Our results suggest that there was no serious immunorejection response when 3D‐BDCS was implanted into the cornea of rabbits, and we can confirm the biocompatibility of 3D‐BDCS by the changes in corneal thickness and the distribution of inflammatory cells. Based on these data, it is expected that 3D‐BDCS can be a promising material for corneal implants.

In ophthalmological point of view, the biocompatibility of the transplanted 3D‐BDCS can be confirmed through the overall thickness reduction of corneal layer after few weeks of the surgery, which can only be verified precisely via dissections. According to the acquired OCT results during biweekly monitoring process, mean corneal thickness value at 4 weeks decreased by around 5.7% compared with the measurements at 2 weeks, which reveals a clear correlation with the examined histological analysis. These results indicate that corneal edema caused by the surgical operation decreased over time, indicating the reduction of foreign body reaction, which showed a similar tendency to that in a previously published article 47. In addition, the histological results with few inflammatory cells clearly support this supposition.

## 4 CONCLUSIONS

We performed the monitoring of 3D‐BDCS‐implanted rabbit corneas in vivo for 1 month using a swept‐source OCT technique at a wavelength of 1310 nm. Based on the high resolution of OCT, we were able to assess the minute morphological changes in rabbit corneas in which 3D‐BDCS had been implanted. Variations in cornea thickness were quantitatively measured in vivo through image processing based on depth‐intensity profiling during the monitoring. To further confirm the biocompatibility of the 3D‐BDCS with corneal specimens, a histological analysis was performed by extracting the rabbits' eyes post mortem and staining the tissues with H&E. Through the results of our investigation of the changes in corneal thickness and the distributions of inflammatory cells, we confirmed the biocompatibility of the fabricated 3D‐BDCS. Although OCT‐based anterior segment imaging has been performed numerous times, the conceptual breakthrough of the study was the development of 3D‐BDCS and non‐invasive biocompatibility confirmation of transplanted cornea through a bi‐weekly monitoring process using SS‐OCT in vivo. As the cross‐sections and enface visualizations were acquired with sufficient resolution and depth visibility, currently existing SS‐OCT engine‐based imaging techniques were used to conduct the study.

As a future study, evaluations of corneal transparency and stiffness could be conducted to confirm that 3D‐BDCS can be used in artificial corneas. Moreover, ultrahigh‐resolution optical coherence microscopy for evaluation such as measuring the 3D‐BDCS thickness is expected to provide a better research environment for researchers in various biomedical and tissue engineering areas.

## AUTHOR BIOGRAPHIES

Please see Supporting Information online.

## Supporting information


**Author Biographies**
