# Supplementary material for: Biocompatibility evaluation of bioprinted decellularized collagen sheet implanted in vivo cornea using swept‐source optical coherence tomography
Source: J Biophotonics. 2019 Jul 23;12(11):e201900098. doi: 10.1002/jbio.201900098 (PMC7065634; doi:10.1002/jbio.201900098)
Supplement: Supplementary file 1 — Author Biographies [file JBIO-12-e201900098-s001.docx]

| 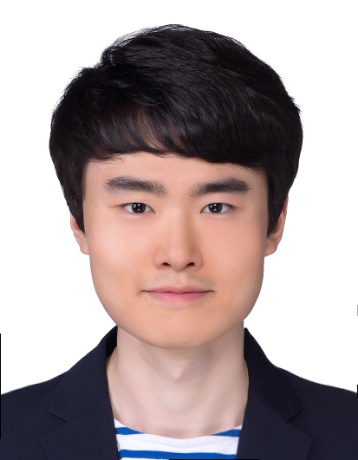 | **Jaeseok Park** is received M.S degree in electronics engineering from Kyungpook National University, Daegu, South Korea, in 2019. His research area is the development of biomedical imaging system, including optical coherence tomography, photoacoustic tomography and their clinical applications. |
| --- | --- |
| 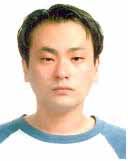 | **Kyoung-Pil Lee** received the Ph.D degree in pharmacology from Pusan National University, Busan, South Korea, in 2016. He is currentel a Research Assistant professor with Kyungpook National University Hospital Bio-Medical research Institute. His research area is the tissue engineering including the retina Microfluidic chip model, Artificial Cornea and scaffold with Nanofiber membranes. |
| 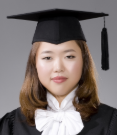 | **Hyeonji Kim** received B.S degree in mechanical engineering from POSTECH, Pohang, South Korea, in 2013. She is currently pursuing the Ph.D. degree in mechanical engineering from POSTECH. Her research area is the development of bioengineered cornea and their clinical applications. |
| 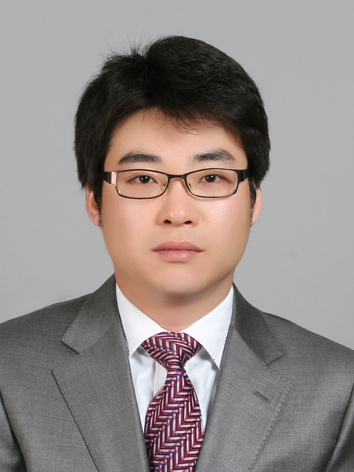 | **Sungjo Park** received the Ph.D. degree in electronics engineering from Kyungpook National University, Daegu, South Korea, in 2015. He was a Research Assistant Professor with the department of Creative IT Engineering, POSTECH. He is currently as Principal Researcher with the Laser Application Center, Kyungpook National University. His research interests are in the development of nonionizing and noninvasive novel biomedical imaging techniques, including photoacoustic tomography, photoacoustic microscopy, optical coherence tomography, and other novel applications of laser processing. |
| 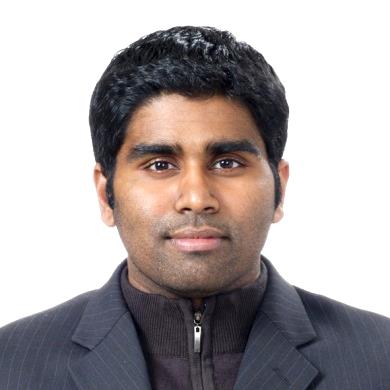 | **Prof. Ruchire Eranga Wijesinghe** received the B.Sc. and Ph.D. degrees in electronics engineering from Kyungpook National University, Daegu, South Korea, in 2012 and 2018, respectively. He is currently an Assistant Professor with the Department of Biomedical Engineering, Kyungil University. His research interests are in the development of high-resolution novel biological and biomedical imaging techniques including optical coherence tomography and microscopy for clinical utility. |
| 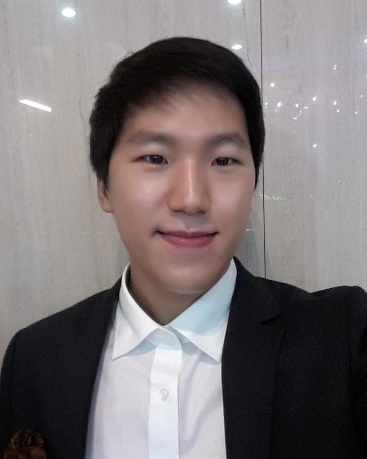 | **Jaeyul Lee** is currently pursuing a Ph.D. degree with the School of Electronics Engineering, Kyungpook National University, Daegu, South Korea. His research interests are in the development of high-resolution novel imaging techniques and optical imaging techniques including photoacoustic microscopy, optical coherence tomography, handheld instruments, and their biomedical applications. |
| **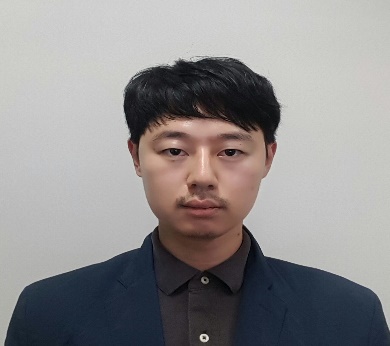** | **Sangyeob Han** is currently pursuing the Ph.D. degree with the School of Electronics Engineering, Kyungpook National University, Daegu, South Korea. His research background includes optical imaging techniques, photoacoustic microscopy, optical coherence tomography and Multiphoton microscopy. |
| 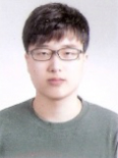 | **Sangbong Lee** received the B.E. degree in electronics engineering from Kyungpook National University, Daegu. South Korea, in 2018. He is currently a MS Researcher with the Electronics Engineering Department, Kyungpook National University. His research interests are development of optical system for biomedical application, and biomedical imaging, including optical coherence tomography, photoacoustic microscopy. |
| **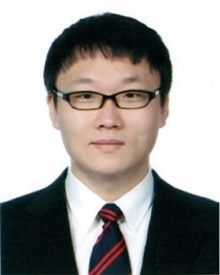** | **Prof. Pilun Kim** received the Ph.D. degree from the Department of Medical and Biological Engineering, Kyoungpook National University, in 2011. He is currently a Researching Visiting Professor with the Institute of Biomedical Engineering, Kyungpook National University. He is interested in translating new technologies from the research field to the application field, such as clinic and industrial and making its productization. His main interests are biomedical device development, optical coherence tomography, and digital image processing. |
| [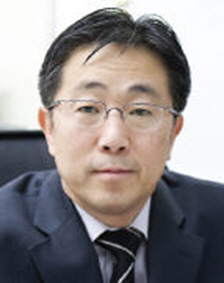](http://www.google.co.kr/url?sa=i&rct=j&q=&esrc=s&source=images&cd=&cad=rja&uact=8&ved=2ahUKEwj9vOCOrPngAhUIQLwKHRf2CUQQjRx6BAgBEAU&url=http://ibio.postech.ac.kr/bbs/board.php?bo_table%3Dm5_11%26wr_id%3D20&psig=AOvVaw2KgBWBI6TGt6T6IZnnEQRW&ust=1552368091655637) | **Prof. Dong-woo Cho** received his Ph.D. in Mechanical Engineering from the University of Wisconsin-Madison in 1986. Ever since, he has been a professor of Department of Mechanical Engineering at the Pohang University of Science and Technology. He is director of the Center for Rapid Prototyping-based 3D Tissue/Organ printing. His research interests include 3D microfabrication based on 3D Printing technology, its application to tissue engineering, and more generally to bio-related fabrication. He has recently focused on tissue/organ printing technology and development of high-performance bio-inks. |
| 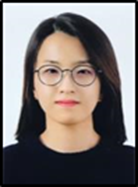 | **Prof. Jinah Jang** received her PhD in Division of Integrative Biosciences and Biotechnology at POSTECH (2015), and worked as postdoctoral fellow in Department of Mechanical Engineering at POSTECH (2015-2016) and Institute for Stem Cell and Regenerative Medicine/Department of Pathology and Bioengineering at University of Washington (2016-2017). She has joined the POSTECH in the spring of 2017 as an assistant professor in the Creative IT Engineering and School of Interdisciplinary Bioscience and Bioengineering, and affiliated in the Mechanical Engineering since 2019. Her research interest lies on the building the functional human tissues from stem cells via the 3D bioprinting technology and printable biomaterials. |
| [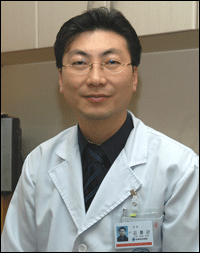](http://www.google.co.kr/url?sa=i&rct=j&q=&esrc=s&source=images&cd=&cad=rja&uact=8&ved=2ahUKEwi3wsr8n_ngAhVkFqYKHbL2AYIQjRx6BAgBEAU&url=http://www.dailian.co.kr/news/view/174740&psig=AOvVaw0-7Cqar-tAi5W5IESNNCvm&ust=1552364880367210) | **Prof. Hong Kyun Kim** received his MD. in 1995 and PhD. in 2007 from School of Medicine, Kyungpook, National University, Daegu, Republic of Korea. He is currently a professor of the Department of Ophthalmology, School of Medicine at Kyungpook National University. He is the current chairman in the department. His research interests are cornea and ocular surface disease, regenerative medicine, and its ophthalmic applications. |
| **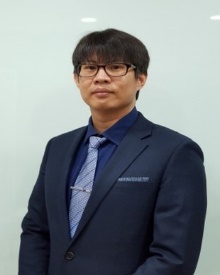** | **Prof. Mansik Jeon** received his PhD in electronics engineering from Kyungpook National University, Daegu, Republic of Korea, in 2011. He is currently an assistant professor of the School of Electronics Engineering at Kyungpook National University. His research interests are in the development of nonionizing and noninvasive novel biomedical imaging techniques, including photoacoustic tomography, photoacoustic microscopy, optical coherence tomography, ultrasonic imaging, handheld scanner, and their clinical applications. |
| **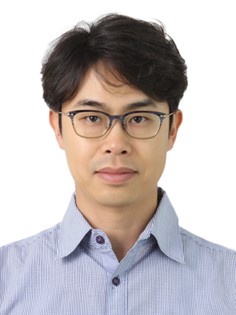** | **Prof. Jeehyun Kim** received his PhD in Biomedical engineering from University of Texas at Austin, USA in 2004. He has worked as a Postdoctoral researcher in University of California, Irvine, at Beckman Laser institute. He is currently an associate Professor at Kyungpook National University, Daegu, Republic of Korea. His research interest is in Biomedical imaging and sensing, Neuroscience studies using Multiphoton Microscopy, Photo-Acoustic imaging and other novel applications of sensors. |
